# Discordant Effects of Putative Lysine Acetyltransferase Inhibitors in Biochemical and Living Systems

**DOI:** 10.3390/cells8091022

**Published:** 2019-09-02

**Authors:** Ryan A. Henry, Yin-Ming Kuo, Zarek S. Siegel, Timothy J. Yen, Jennifer Rhodes, Erika A. Taylor, Andrew J. Andrews

**Affiliations:** 1 Department of Chemistry and Biochemistry, Wilkes University, 84 West South Street, Wilkes-Barre, PA 18766, USA; 2 Cancer Epigenetics, Fox Chase Cancer Center, 333 Cottman Avenue, Philadelphia, PA 19111, USA; 3 Chemistry Department and Molecular Biophysics Program, Wesleyan University, 45 Wyllys Ave, Middletown, CT 06459, USA; 4 Cancer Biology, Fox Chase Cancer Center, 333 Cottman Avenue, Philadelphia, PA 19111, USA; 5 Blood Cell Development and Function Program, Fox Chase Cancer Center, 333 Cottman Avenue, Philadelphia, PA 19111, USA

**Keywords:** p300, KAT, drug screen, epigenetics, histones, mass spectrometry

## Abstract

Lysine acetyltransferases (KATs) are exquisitely fine-tuned to target specific lysine residues on many proteins, including histones, with aberrant acetylation at distinct lysines implicated in different pathologies. However, researchers face a lack of molecular tools to probe the importance of site-specific acetylation events in vivo. Because of this, there can be a disconnect between the predicted in silico or in vitro effects of a drug and the actual observable in vivo response. We have previously reported on how an in vitro biochemical analysis of the site-specific effects of the compound C646 in combination with the KAT p300 can accurately predict changes in histone acetylation induced by the same compound in cells. Here, we build on this effort by further analyzing a number of reported p300 modulators, while also extending the analysis to correlate the effects of these drugs to developmental and phenotypical changes, utilizing cellular and zebrafish model systems. While this study demonstrates the utility of biochemical models as a starting point for predicting in vivo activity of multi-site targeting KATs, it also highlights the need for the development of new enzyme inhibitors that are more specific to the regulation of KAT activity in vivo.

## 1. Introduction

Histone post-translational modifications play a major role in regulating access to DNA, and as such are important in the regulation of gene transcription and DNA repair. Due to this biological role, a significant amount of research focuses on creating a better understanding of the proteins that regulate histone modifications. One such modification, histone acetylation, is regulated by lysine acetyltransferases (KATs, also referred to as histone acetyltransferases (HATs)) and histone deacetyltransferase (HDACs). One of the most studied KATs, p300, is a prolific acetyltransferase, with mutations in p300 resulting in several diseases including neurological disorders and cancer [1,2,3,4,5]. Because of this association, correcting aberrant levels of acetylation could prove instrumental in treating the diseases associated with them.

There are two major obstacles to better understanding the role of histone acetylation in maintaining a healthy cellular state. The first is the sheer number of potential sites of acetylation on histones: Nucleosomes are made up of an octamer, consisting of two copies each of histone H2A, H2B, H3, and H4. On this octamer there are dozens of lysine residues that have been shown to be targeted for acetylation in a biological context. Additionally, each KAT demonstrates specificity for particular lysine residues, emphasizing the importance of understanding the mechanisms underlying site-specific histone modification. This first obstacle can be addressed by utilizing mass spectrometry analysis, which allows the simultaneously quantitation of modification to multiple lysine residues [6,7].

The second major obstacle to investigations into the biological impact of histone acetylation is the quality of currently available small-molecule regulators of histone acetyltransferases, and p300 in particular. For this investigation, we studied several putative p300 targeting compounds, including EGCG, CTPB, CTB, and garcinol (described below), while also continuing our characterization of C646, which we have previously studied [7]. Epigallocatechin gallate (EGCG) is a phenolic antioxidant found in green tea, and has been shown to affect p300 acetylation of several non-histone proteins including RelA (p65) [8]. However, EGCG also targets a number of other proteins in addition to KATs within the cell [9], making it non-specific for regulating p300. Garcinol is a lysine acetyltransferase inhibitor, known to affect various lysine acetyltransferases, including p300, pCAF, and Gcn5 [10,11]. Unfortunately, this lack of inhibitory selectivity among several different members of an enzyme family that harbors distinct active sites suggests a promiscuous inhibitory mechanism, potentially as an aggregator [12]. Additionally, EGCG has been recognized as a Pan Assay Interference Compound (PAIN) [13], with the catechol component of garcinol potentially behaving in a similar manner. CTPB (*N*-[4-chloro-3-(trifluoromethyl)phenyl]-2-ethoxy-6-pentadecylbenzamide) was classified as the first known small molecule activator of p300 [14], while CTB (*N*-(4-Chloro-3-trifluoromethyl-phenyl)-2-ethoxy-benzamide) is a simplified analogue of CTPB [15]. CTB is more cell permeable, as opposed to CTPB which is characterized by low cell permeability [16]. Finally, C646 was initially characterized as a small molecule inhibitor of p300 that functions by competing with acetyl-CoA for binding to the enzyme, although previous work from our lab has shown that C646 has a biphasic effect on p300 activity [7,17]. 

For these reasons, many of these small molecules are not ideal compounds for the modulation of histone acetylation. Indeed, recent studies have been undertaken to further pinpoint the off-target effects of reported histone acetyltransferase-targeting drugs compounds [18]. Despite these issues, these compounds are still widely utilized in the field as investigative tools, with greater than 500 references in 2016 alone. Promisingly, though, this problem has begun to be recognized by the field, and current inroads have been made to develop compounds that more specifically target KATs like p300 while minimizing potential off target interactions [19]. However, it would be useful to develop tools that accurately predict the activity and specificity of small molecules that target KATs. Here, we present a multifaceted investigation for systematically identifying the agreements and conflicts among methods for these inhibitors using integrated in silico (e.g., docking), in vitro (e.g., kinetic assays), and in vivo (e.g., zebrafish embryos) approaches. By employing these combined approaches, we overcome the intrinsic limitation of each individual method, and are able to identify biologically relevant phenomena while building mechanistic models for understanding the activity and specificity of KAT-targeting compounds.

Thus, this work can serve as a template for studying the effects of potential histone acetylation-altering compounds, help to identify potential issues with these compounds, and provide valuable information on their in vitro and in vivo activity. Importantly, the methodology we have developed can be utilized to help address the lack of highly specific KAT-targeting compounds by providing a rapid and efficient means for identifying and testing novel p300-targeting drugs in the future.

## 2. Materials and Methods

**Reagents**—All Chemicals were purchased from either Sigma-Aldrich (St. Louis, MO, USA) or Fisher Scientific (Waltham, MA, USA); the purity was the highest commercial grade available or met LC/MS grade. Ultrapure water was generated from a Millipore Direct-Q 5 ultrapure water system (Burlington, MA, USA). Recombinant histone H3 and H4 were purified and provided from the Protein Purification Core at Colorado State University. Acetyl-CoA was obtained from Sigma-Aldrich. Garcinol and CTPB were purchased from Abcam (Cambridge, MA, USA), CTB from Sigma-Aldrich, EGCG from Cayman Chemical, and C646 from EMD Millipore.

**Protein purification**—Full length p300 was expressed and purified from Sf9 cells as previously described [20]. The p300 construct was graciously provided by Karolin Luger (Colorado State University). 

**Kinetic experiments**—Experiments to characterize the effect of various drugs on p300 acetylation were conducted under k_cat_ conditions in buffer containing 100 mM Ammonium bicarbonate and 50 mM HEPES buffer (pH 7.8) at 37 °C. All reactions were performed in the presence of 0.2% DMSO. Experiments were performed utilizing excess H3/H4 (15 µM) and acetyl-CoA (200 µM) in the presence of p300 (5 nM). A time course was performed to determine the rate (v/E) of acetylation. Assays were quenched using four volumes of trichloroacetic acid (TCA), and prepared for mass spectrometry analysis as previously described [20]. The rate of acetylation was then determined utilizing varying concentrations of drug (EGCG, CTB, CTPB, Garcinol, or C646) 

**Measurement of acetylation from cell culture samples**—Histones were extracted overnight from cell pellets using 0.2 N HCl. Histones were precipitated with trichloroacetic acid (TCA), and prepared for mass spectrometry analysis as previously described [20]. Quantification of acetylation marks was performed using mass spectrometry analysis, as outlined below. 

**Treatment of zebrafish embryos**—Embryos were treated 0.5 h post fertilization with either 0.2% dimethylsulfoxide (DMSO) or the indicated dose of C646, EGCG, CTB, CTPB, or garcinol. Groups of 110–130 embryos were arrayed into six-well plates with a total of 1.5 mL of E3 zebrafish embryo media. For histone extraction, embryos were washed with ½ x Ringer’s solution, before being lysed with Triton extraction buffer (PBS containing 0.5% Triton X 100 (*v*/*v*), 0.02% (*w*/*v*) NaN_3_). Histones were extracted overnight using 0.2 N HCl. 

**UPLC-MS/MS analysis**—A Waters Acquity H-class UPLC (Milford, MA, USA) coupled to a Thermo TSQ Quantum Access (Waltham, MA, USA) triple quadrupole (QqQ) mass spectrometer was used to quantify acetylated H3/H4 peptides as previously described [20,21].

**QqQ MS data analysis**—Each acetylated and/or propionylated peak was identified by retention time and specific transitions, as previously reported [20,21]. The resulting peak integration was done using Xcalibur software (version 2.1, Thermo, Waltham, MA, USA). The fraction of a specific peptide (*F_p_*) is calculated by equation 1, where *I_s_* is the intensity of a specific peptide state and *I_p_* is the intensity of any state of that peptide, and analyzed as previously described [21,22].

(1) $$F_{P}\;=\;I_{S}/(\sum I_{P})$$

**Data analysis**—All models were fit to the data-using Prism (version 5.0d, GraphPad Software, San Diego, CA, USA). The initial rates (*v*) of acetylation were calculated from the linear increase in acetylation as a function of time prior to 10% of the sum of acetylated residues. For comparisons of v/E (in kinetic analysis) or fraction acetylation (in cellular and zebrafish samples), the significance of the change in rate was calculated using Prism, via two-tailed, unpaired t-tests. In vitro drug titrations were fit using a single-phase inhibition or a two phase model where the first is activating and the second is inhibiting (for details on data fitting see Henry et al., 2015 [7]). 

**Computational docking and analysis**—The structure of p300 (PDB 3BIY, 1.7 Å)^1^ was used as a template for docking experiments. Using PyMol^2^ (v1.8, Schrödinger LLC, New York, NY, USA), the bisubstrate analogue inhibitor bound in this structure, Lys-CoA, was split into acetyl-CoA and lysine portions for later binding site analysis. For docking, all bound ligands, including the five bromine atoms and all waters, were removed from the structure. The prepare_receptor4.py script included in AutoDock Tools^3^ (Scripps Research, Jupiter, FL, USA) was used to merge non-polar hydrogens, add Gasteiger charges, and assign AutoDock4 atom types to prepare the protein for docking. Using ChemDraw v14.0 (PerkinElmer, Waltham, MA, USA) followed by the OpenBabel^4^ babel program, the five ligand molecules (Garcinol, CTB, EGCG, CTPB, C646) were prepared as energy minimized 3D models using the MMFF94 force field. 

Using the AutoDock Vina plugin for PyMol*, a grid box was designed to include the bound Lys-CoA (taken as an approximation of the location of the acetyl-CoA and lysine substrates) and all protein atoms within 5 Å. The (x, y, z) dimensions of this box were (−17.63, 15.96, 1.48) for the center and (19.75, 31.6, 19.75) for the size. A second grid box was designed to include the entire protein. The (x, y, z) dimensions of this second box were (−8.38→, 25.49, 1.43) for the center and (126, 126, 126) for the size. Docking was performed using AutoDock Vina^5^ (v1.1.2, (Scripps Research, Jupiter, FL, USA)) on the Wesleyan University High-Performance Computing Cluster. A shell script was written to submit 20 jobs generating 20 conformations each in parallel, for a total of 400 poses for each of the five ligands. The exhaustiveness parameter was set to 50, 2 CPUs were used for each job, with all protein residues kept rigid, but all single bonds in the ligands freely rotatable.

After docking, the resulting poses were analyzed to obtain the number and identity of the close contacts between each ligand and the protein residues, as well as the binding data from each pose. By examining the ligand interactions with residues within 5 Å of either the acetyl-CoA or the lysine parts of lys-CoA analogue, each ligand was assigned to one or more binding site. These data were organized in R and graphed using the ggplot2 package, and the 3-dimensional structures of the results were visualized using PyMol.

We defined regions of the acetyl-CoA pocket on the basis of the binding regions of a lys-CoA molecule. Residues for the Lysine pocket: ARG1627, ASP1444, CYS1438, ILE1395, SER1396, TRP1436, TYR1397, TYR1446; residues for the acetyl-CoA pocket: ALA1437, ARG1410, ARG1462, ASP1399, CYS1438, GLN1455, HIS1451, ILE1395, ILE1435, ILE1457, LEU1398, LEU1463, LYS1407, LYS1456, LYS1459, PRO1439, PRO1440, PRO1458, SER1396, SER1400, THR1411, TRP1436, TRP1466, TYR1397, TYR1414, TYR1446, TYR1467). The non-active site pockets were identified by examining all of the consensus residues within 5 Å of a given ligand, when bound away from the active site. The residues for each of the two pockets are: pocket 1 - SER1329, ASP1330, LYS1331, THR1332, PRO1354, TYR1355, ARG1356, THR1357, LYS1358, GLU1380, TYR1381, GLY1382, SER1383, ASP1384, CYS1385, LEU1428, TYR1430, PRO1611, ILE1612, VAL1613 ASP1614, PRO1615, ASP1616, PRO1617, LEU1618; and pocket 2 - GLU1348, MET1349, ALA1350, PHE1353, PRO1386, PRO1387, PRO1388, ASN1389, GLN1390, ARG1391, ARG1392, THR1431, THR1432, GLY1433, HIS1434, ASP1482, TYR1483, LYS1484, ILE1486, GLN1489, ALA1490, GLU1492, ASP1493, ARG1494, LEU1495, SER1497, LYS1499, GLU1500, LEU1501, PRO1502, VAL1597, ILE1598, ARG1599, LEU1600, ILE1601, ALA1602, GLY1603, PRO1604, ALA1606, ASN1607, SER1608, LEU1609.

## 3. Results

### 3.1. In Vitro Drug Screening to Measure Changes in the k_cat_ of p300

A fundamental understanding of how a compound affects p300 comes from in vitro assays. In order to quantify site-specific changes in p300 histone acetylation activity, we utilized selected reaction monitoring (SRM), a quantitative MS-based approach that enables the simultaneous measurement of acetylation of multiple lysine residues [7,20,23,24].

To characterize p300 histone acetylation activity, we began by measuring the catalytic efficiency of p300 at multiple sites of histone H3/H4; we have previously shown that determining how the site-specific catalytic efficiency (k_cat_) of an acetyltransferase changes in response to external factors, such as drug treatment, can be utilized as a predictor of how that factor will alter the selectivity of a KAT in cells [7,23]. To determine the k_cat_ of p300, time-course assays were performed under steady-state conditions in the presence and absence of our selected drugs. Under steady-state conditions, p300 activity is concentrated at the H3 and H4 tails residues [7,20]. Although other sites of acetylation are reported for p300 [25,26], we do not observe additional acetylation events under these conditions, and thus focused our analysis on 4 key residues of the H3 tail: histone H3 lysine 9 (H3K9), H3K14, H3K18, and H3K23. The k_cat_ values for p300 at these residues in 0.2% DMSO has been previously determined [7], and are included in Table 1 for reference.

**Figure 1:**
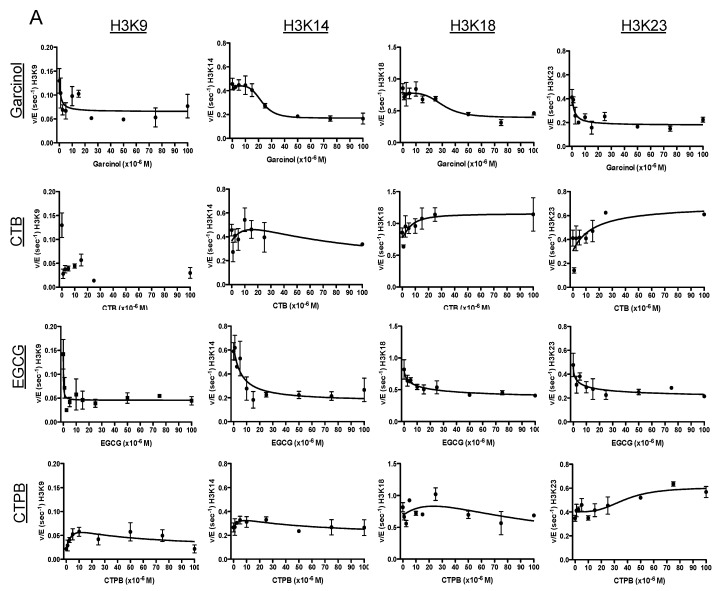

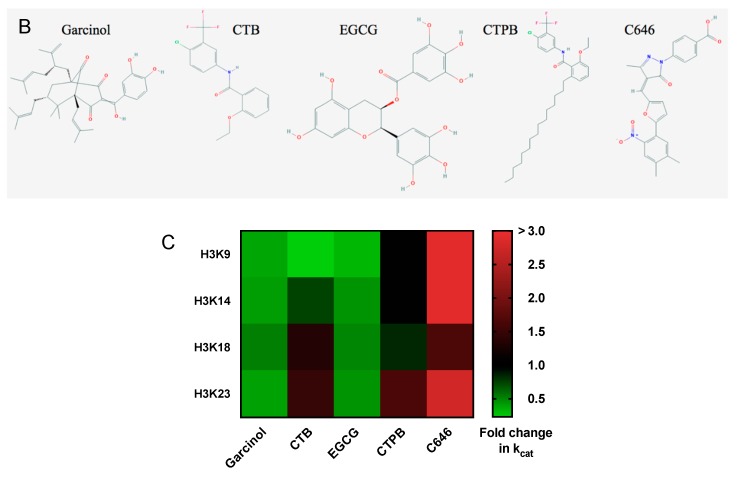
Site-specific characterization of lysine acetyltransferase targeting drugs. (**A**) Changes in p300 acetylation rate under k_cat_ conditions as a result of drug treatment. An in vitro kinetic assay was performed in the presence of p300 and excess H3/H4 and acetyl-CoA. Shown are the rates of acetylation of the tail residues of histone H3 (H3K9, H3K14, H3K18, and H3K23) as a function of drug concentration (from top to bottom: garcinol, CTB, EGCG, and CTP. (**B**). The error bars at each data point represent the v/E value determined from a linear fit of 5 time points. There is no fit line for H3K9 in the presence of CTB as the parameters could not be determined. The initial k_cat_ as well as the fold-change of k_cat_ in the presence of drug are also summarized in Table 1. (**B**) Structures of the five compounds utilized for this study. Images obtained from PubChem [27]. (**C**) Heat map of the fold change in k_cat_ when treated with drug. Includes previously determined values for C646 [7].

When characterizing the effect of these drugs on the k_cat_ of p300 (Figure 1 and Table 1), CTPB was found to have the most focused effect on a single residue of H3: it showed an increase in rate at H3K23 (~1.6-fold), while it had a minimal effect on other sites (H3K9 and H3K14). In comparison, the effect of CTB was also greatest at H3K23 (an increase of ~1.5-fold), but interestingly CTB had more of an effect on acetylation of other sites than CTPB: in the presence of CTB the rate of acetylation at H3K18 increases 1.34-fold, while decreasing to 0.23 and 0.74 at K9 and K14, respectively. Meanwhile, we observed that treatment with garcinol led to a decrease in the rate of acetylation across each site of H3, with the largest decrease at H3K9, which decreased to ~30% of the initial rate. The rates at K14, K18 and K23 were also reduced by >50%. Like garcinol, EGCG also had a strong inhibitory effect on p300 activity, and displayed a similar pattern of activity, resulting in the largest decrease in the rate of acetylation at H3K9. These experiments were also repeated utilizing CBP, a KAT with high sequence homology (59% sequence identity) to p300, as a basis of comparison for the protein specific effects of their activity (Table 1). We have previously reported on the results of similar experiments using the p300-targeting compound C646 [7]. As a point of comparison, a summary of the kinetics parameters found in that paper (as well as the effects of C646 in cells) are included in Table 1. Each of these experiments provides a baseline to establish how, under ideal conditions, the selected compounds will affect p300 activity. However, as the purpose of this investigation is to provide a multi-faceted view of the effects of these compounds, further experimentation was performed to determine how these drug responses may be altered in a more complex environment, such as treatment of cells in culture.

### 3.2. Screening Changes in Acetylation in Response to Drug Treatment in Cells

In order to better understand how the in vivo activity of these drugs compared to the characterized in vitro activity, we next determined how these drugs function in a cellular context. In addition to correlating the in vitro results with cellular data, for the development of a methodology for characterizing histone acetylation-altering drugs, this step serves as a benchmark for eliminating potential drugs from a screen before moving into an animal system. There are a number of reasons why the information gained from the in vitro data might vary in a cellular system: p300 is not the only KAT present in a cellular system, and thus solely affecting its activity might not cause the level of changes in cells that were observed in vitro; there could be off-target effects of these compounds on other KATs; other proteins in the cell interacting with p300 could affect drug interaction with p300, or alter drug efficiency; binding of the compound to other proteins could potentially affect its efficacy for altering histone acetylation; finally, the ability to deliver the drug inside of a cellular system (cell permeability and/or solubility) cannot be tested in vitro.

**Figure 2:**
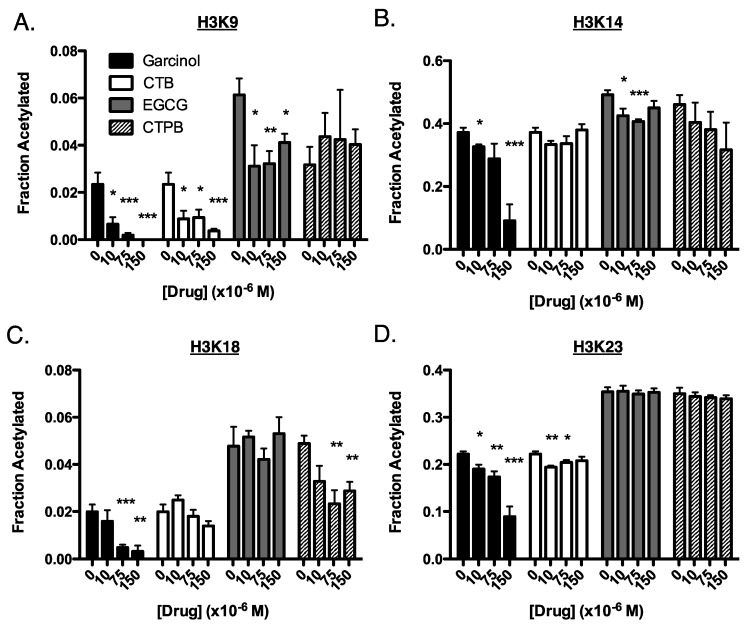
Site-specific changes of histone acetylation in BxPC3 cells in response to drug treatment. Cells were treated for 4 h with 0, 10, 75, or 150 µM of either garcinol (black bars), CTB (white), EGCG (gray), or CTPB (striped). Shown are the fraction of acetylation of the tail residues of histone H3: (**A**) H3K9 (**B**) H3K14, (**C**) H3K18, and (**D**) H3K23. Experiments were performed in triplicate (3 replicates within the same experiment). Data are represented as mean ± SEM. These results are also summarized in Table 1. (*) *p* < 0.05, (**) *p* < 0.005, (***) *p* < 0.001 when compared to the untreated control.

For these experiments, we performed a titration of each drug in BxPC3 cells (human pancreatic cancer), which we used previously to test C646, as BxPC3 cells have no reported mutations in p300 nor CBP [7]. We measured the level of acetylation in cells before treatment and 4 h post-treatment (as we previously noted a peak in effect on acetylation after 4 h) [7]. As with our in vitro data, we found the greatest change in histone acetylation in response to garcinol, where there was a general decrease in acetylation at each site of H3 (Figure 2 and Table 1). However, a number of deviations in the response to drug treatment in BxPC3 cells can be seen when compared to the kinetics information gained in vitro. For example, while treatment with EGCG lead to a decrease in acetylation at H3K9 and H3K14 both in vitro and in cells, in vitro there was also a drop in acetylation at H3K18 and H3K23 that is not observed in cells. In cells, CTPB showed only modest changes in acetylation, most strongly at H3K18, which was in line with in vitro results, but in vitro CTPB caused an increase in H3K23 acetylation, a response not found in cells. CTB had a stimulatory effect in vitro at H3K18 and H3K23 which were not observed in cells, although both systems saw the greatest decrease in acetylation at H3K9. 

These results provide an important picture of the discrepancies that can be seen when screening the effect of KAT targeting drugs in vitro versus in cells, highlighting the importance of testing compounds in various systems, and providing a better biological context to the actual efficacy of such drugs. Thus, to further investigate the response to these drugs in a biological context, we continued our investigation using a zebrafish model system. 

### 3.3. Testing for Drug Lethality in Zebrafish Embryos

**Figure 3:**
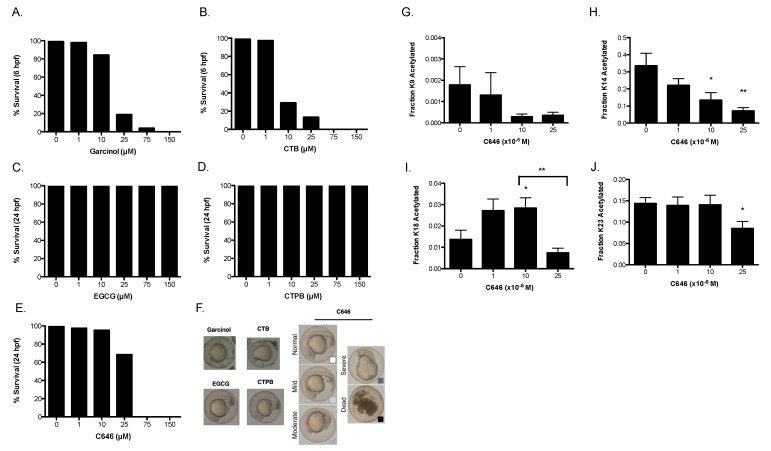
Drug treatment of zebrafish embryos. Zebrafish embryos were collected and treated for 24 h with drug. The survival rates at 6 h for (**A**) garcinol and (**B**) CTB, 24 h for (**C**) EGCG, (**D**) CTPB, and (**E**) C646 were measured. (**F**) Representative pictures of the phenotypes found at these time points. Histone acetylation levels of embryos treated with C646 were measured at 24 h. Shown are the fraction of acetylation at (**G**) H3K9, (**H**) H3K14, (**I**) H3K18, and (**J**) H3K23. This experiment was performed in triplicate, and data are represented as mean ± SEM. (*) *p* < 0.05, (**) *p* < 0.005 when compared to the untreated control, unless the comparison is otherwise indicated.

We next tested how treatment with these drugs affects the biology of a healthy organism. While it is possible that drug treatments will alter the biology of cells in culture, changes were not obvious and would require more extensive molecular analysis. To further expand on our in vitro and cellular findings, we tested for survival and phenotypic changes in zebrafish embryos (Figure 3). These experiments were intended to accumulate data that would allow us to draw connections between changes in histone acetylation and in vivo effects as a predictive tool to find a safe dosage for drug treatment. 

Embryos were exposed to drug at the 1-cell stage and survival was scored at 6 and 24 h post-fertilization (hpf). Groups of embryos contained 110–130 embryos arrayed on a 6 well plate. Phenotypes listed in Table 1 are based on the most abundantly observed phenotype from each treatment group. These phenotypes were sorted into broad categories, of either normal (mostly healthy), moderate (slight deformations or abnormal growth), severe (significant deformations) or dead (the embryo did not survive), although it is worth noting that under none of the conditions was “severe” the most abundant phenotype, as death of the embryo was likely at that point.

We found that the embryos treated with up to 25 µM C646 survived up to 24 hpf, while causing death at higher concentrations. Treatment with garcinol and CTB at 25 µM and 10 µM respectively, resulted in death earlier, at 6 hpf. At 24 hpf, 10 µM of garcinol was also causing widespread embryonic lethality, although 1 µM CTB and Garcinol was well tolerated at 24 h (data not shown). Meanwhile, nearly 100% of the embryos survived treatments with CTPB and EGCG. As the intention of collecting this data was to make connections between survival and changes in histone acetylation, we noted that decreases in H3K9 acetylation levels observed in cells correlated closely with embryo death (see Discussion). 

To determine the feasibility of directly measuring histone acetylation changes in the embryos, we focused on C646 treatment, performing acid extraction of histones from pools of control- or C646-treated embryos at 24 hpf. These samples were also analyzed using our mass spectrometry-based method (Figure 3G–J). Consistent with the lower levels of basal H3K9 acetylation in our cell culture data, very little acetylation of H3K9 occurred in zebrafish. Despite basal levels of acetylation being below 0.2%, we could see a discernable decrease in acetylation with the addition of C646 (Figure 3G). Additionally, we found that treatment with C646 led to a dose dependent decline in H3K14 acetylation (Figure 3H). H3K18, meanwhile, displayed the biphasic change that we have previously reported from C646 treatment in cells (Figure 3I) [7]. Finally, while H3K23 acetylation remained largely unchanged at lower concentrations of C646, it was inhibited at higher concentrations (25 µM) of C646 (Figure 3J). These results validate our cell culture data and in vitro kinetic data and also demonstrate the feasibility of monitoring changes in histone acetylation directly in zebrafish embryos with our MS approach.

### 3.4. In Silico Docking of p300 Interacting Drugs

**Figure 4:**
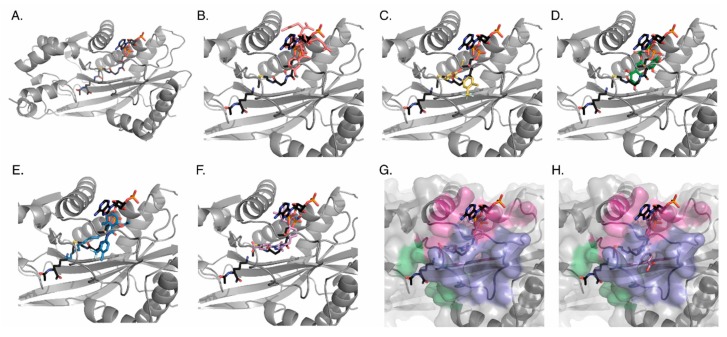
In silico docking of compounds to p300. Each ligand was docked to the active site of the p300 KAT domain 400 times to generate a series of poses. For each ligand image, lysine and acetyl-CoA binding geometries (Charcoal) are provided as reference, and the tightest binding geometry of each ligand is shown. (**A**) The whole crystal structure of p300 with only lys-CoA bound (PDB 3BIY; gray). Close up of the acetyl-CoA binding pocket with (**B**) Garcinol (salmon), (**C**) CTB (yellow), (**D**) EGCG (green), (**E**) CTPB (blue), or (**F**) C646 (lavender) bound. (**G**) and (**H**) Surface map depiction illustrating that while the ligands predominantly occupy the lys-CoA tunnel, the ligands also occupy an additional previously undescribed side tunnel: (**G**) C646 bound in primary tunnel (**H**) C646 bound in side tunnel. All compounds were also docked to the entire protein allowing identification of two additional binding sites outside of the active site.

In silico modeling is a popular form of drug screening, with several p300 targeting compounds discovered via this type of analysis. As such, we were interested in determining whether the observed effects of p300 targeting drugs correlated in anyway with where and how they bind to p300. Thus, we performed in silico docking experiments to determine compound interaction with the catalytic portion of the KAT domain. Though the KAT domain of p300 represents only 16% of the overall structure, it is catalytically active in the absence of other domains [28,29]. To simulate binding of target drugs to p300, we performed in silico docking experiments using Autodock Vina [30] for these 5 compounds to both the active site of the p300 KAT domain and also the entire KAT domain of p300. 400 ligand poses were generated for both conditions and analyzed (as described in Methods) for both binding affinity and binding location (Figure 4). 

When constraining the docking to the active site, all of the ligands bound in a competitive fashion with either histone, acetyl-CoA, or both. To quantitate this observation, the tunnel that defines the active site was divided into two different regions: the histone lysine pocket and the acetyl-CoA pocket. We defined regions of the acetyl-CoA pocket on the basis of the binding regions of a lys-CoA molecule (see Methods) (Figure 4A). Careful examination of the binding poses revealed that some of the ligands also occupied a previously undescribed side tunnel in the acetyl-CoA site (Figure 4H); residues of this side pocket are: ASP1399, ASP1444, ASP1445, ASP1454, CYS1450, GLN1455, GLU1442, GLY1443, HIS1451, ILE1447, ILE1457, PHE1448, PRO1440, PRO1452, PRO1453, SER1441, TYR1446). We quantitated the number of ligand poses observed within the three pockets, as well, and also the energy distribution of the ligands in each region (Supplemental Figure S1A,B). The data revealed average binding energy for all compounds between −6 to −10 kcal/mol, with values of −6.942, −7.556, −7.898, −7.228, and −9.799 for Garcinol, CTB, EGCG, CTPB, and C646, respectively. 

When each of these compounds was docked to the entire KAT domain of p300 we observed that all of the C646 poses bound in the active site, while greater than 45% of the ligand poses for Garcinol and EGCG were in pockets on the opposite face from the active site (see Methods for residue numbers) (Figure 4 and Supplemental Figure S1C). While it is unclear if there is a physiological function of these distal pockets, residues located within each of them include residues previously associated with cancer mutations in p300 according to analysis with COSMIC (Allo pocket1: PRO1354, ARG1356, and CYS1385; Allo pocket2: ALA1350, ALA1350, and ARG1599) [31].

While these in silico binding experiments are a valuable tool for identifying compounds that bind to p300, data obtained from docking to the active site (either the binding position or variation in affinity frequency) failed to accurately predict in vitro or in vivo (in cells) specificity changes in the presence of drugs. However, the docking to the entire KAT domain of p300 revealed that selective in silico active site binding was associated with in vivo inhibition of p300. 

## 4. Discussion

A collection of highly specific and effective regulators of lysine acetyltransferases are currently lacking, limiting our ability to accurately probe the effects of histone modification independent of other off-target effects. Contributing to this problem are the unique challenges presented by multi-targeting enzymes, which hinder rapid and accurate identification of the compounds affecting them. Here we have investigated the efficacy of several previously identified p300-targeting compounds, while using this investigation as a mechanism for outlining a combined methodology for a rapid screen for classifying KAT-targeting compounds. This method begins with a k_cat_ analysis in vitro, as an initial indication of the efficacy and site-specific effects of the drugs on histone acetylation. Subsequent experiments in cells allow us to further characterize these compounds in a more complex biological environment, allowing us to identify discrepancies between in vitro and in vivo behavior. The use of a zebrafish model also allows us to determine tolerable dosage ranges for these drugs and potential deleterious effects of the screened compounds. Utilizing increasingly complex models ensures the most efficient and effective use of resources, while providing valuable information about the drugs at each stage. By identifying relevant compounds and providing a steady stream of data to create meaningful connections between kinetics, cellular data, and in vivo treatment, this methodology provides a means for continuous discovery and characterization of novel molecular biology tools. 

As we would predict, we have found that drugs that are more specifically targeted to KATs have a closer relationship between their in vitro characterization and the changes in acetylation they bring about in cells. For example, we have seen in the past that the changes seen with C646 in vitro closely mimic the trends seen in cell culture [7]. This compound was specifically designed to bind and affect p300 and the homologous CBP, and thus we can more closely predict the biological response to the compound with our in vitro experiments. Consistent with its design, the compound was shown to have the greatest calculated binding affinity for p300 among all the compounds tested herein, with half of the compounds binding in poses with binding affinities below −10 kcal/mol. Further, with our zebrafish model, we observed a similar biphasic activity at H3K18 as observed in vitro. Garcinol exhibits an inhibitory effect in both in vitro and in cells; however, the decrease in histone acetylation caused by garcinol is actually greater in cells than in vitro. This is likely due to the fact that garcinol is known to affect several KATs, including p300, CBP, and PCAF. In silico binding shows moderate binding affinity and significant competition with the CoA portion of the binding pocket when docked against the active site. Since CoA recognition motifs are generally conserved between enzymes, this result is consistent with the observed inhibition promiscuity. As previously mentioned, this lack of inhibitory selectivity suggests a promiscuous inhibitory mechanism, such as aggregation [12], which is supported by the propensity for binding poses to localize on the surface of the KAT domain away from the active site. Meanwhile, EGCG, which has been shown to have a wide variety of binding partners (including DNA and RNA [32], proteasomes [33], and T-cells [34], to name a few) has less of an effect on histone acetylation in vivo than when combined solely with p300 in vitro. EGCG was also noted for binding to sites away from the active site, which would also have a confounding effect on translating results from in vitro to in vivo. CTPB also has a lesser response in cells compared to in vitro, likely due to its characterized low cell permeability [35]. These compounds also had intermediate in silico affinity for p300, and a small number of poses found outside of the active site.

Recently, significant effort has been put into developing compounds that are more specific for p300 (and CBP). For example, a publication by Lasko et al. outlines a methodology for developing a highly specific p300 and CBP inhibitor. In this investigation, the authors also utilize a multifaceted approach in order to design and test a specific inhibitor: Their process starts with a computer-based virtual ligand screening, followed by in vitro and in vivo testing. Doing so, they are able to generate a compound that is highly specific to p300 and CBP. This type of investigation highlights the importance of the type of methodology we outline here, one which utilizes multiple systems to test and classify purported acetyltransferase targeting compounds. This type of repeated and varied testing, as we suggest here, is key to avoiding false hits that may appear in either in vitro, in silico, or even in vivo experiments when performed alone.

In addition to outlining an efficient screen for KAT-targeting compounds, our results emphasize the importance of understanding changes in selectivity of multi-targeting enzymes. We see, both in vitro and in vivo, that the tested compounds consistently alter histone acetylation in a site-dependent manner (Table 1), and neither global acetylation levels nor assessment of acetylation levels at a single site provides a robust account of drug effect. This is especially true for drugs like CTB, that display contrary actions on different residues: In such a situation, probing for global levels of histone acetylation can be misleading as drugs may seem ineffective, or large changes at a single site could be masked by multiple small changes at other sites. Therefore, we create a more accurate understanding of drug function via multi-site analysis.

Although the specificity of the tested compounds has largely proven to be subpar, they may have provided us with an interesting investigative lead that deserve further attention: Comparing our cell data with our zebrafish data, we noted that decreases in H3K9 acetylation levels observed in the cellular work (Figure 2) correlated closely with death in the zebrafish embryo model (Figure 3). CTB, C646, and garcinol all have a large effect on K9 acetylation, decreasing levels to less than 25% of the control. The fact that EGCG has a decrease to 59% of the control without affecting survival would suggest that there is a minimum threshold that must be crossed before leading to embryonic death. The importance of H3K9ac in development is emphasized by using compounds with overlapping effects on H3K9ac but not in other areas (for example, garcinol decreases H3K18 acetylation, while C646 increases acetylation of this site). Our quantitation of H3K9ac in zebrafish has shed some light on this result, as we noted that H3K9ac levels are generally very low in the zebrafish embryos to begin with (less than 0.5% acetylated) (Figure 3G). This could account for the very high sensitivity, and lethality, to H3K9 altering drugs. While these low levels of H3K9 acetylation in zebrafish embryos make quantitating subtle changes in acetylation difficult in embryos, this highlights the utility of using multiple model systems for our screen, as this type of multi-step analysis allows us to create a more complete picture by making connections between what we observe in vitro, in cells, and in embryos. It would be worthwhile to continue this investigation once more specific KAT targeting compounds can be identified.

Finally, of note, using the KAT domain of p300, we have shown that the in silico binding affinity can broadly predict whether p300 inhibition will correlate with in vitro and in vivo results–whereby the compounds with the highest affinity and that target the active site selectively (when docked against the entire protein) will correlate most strongly. Additionally, this study showed multiple different binding modes of our screened drugs, and identified distinct regions within the acetyl-CoA binding pocket for these small molecules to bind (Figure 4). We have elucidated a previously uncharacterized side-pocket within the acetyl-CoA binding pocket that has potential use in future drug discovery and design. We have also potentially identified regions of the protein distant from the active site that maybe important to the function of p300. While this in silico investigation corroborated our other studies, the lack of structural information for the complete p300 protein likely prevented the docking from being more predictive. This further highlights the problems associated with highly confined docking experiments and the necessity of augmenting the data obtained from in silico screens using the outlined multi-system analysis. As we continue to grow our screen to include new and uncharacterized small molecules, we hope to elucidate whether different binding modes and occupation of binding regions identified in in silico screens can be used to predict how compounds differentially affect p300 activity.

In summary, we have demonstrated discrepancies in the in vitro, in vivo and in silico methods for predicting efficacy of several putative p300-targeting compounds. We have worked to generate a multi-faceted approach to more accurately identified the biological impact of compounds that target lysine acetyltransferases. We have shown an efficient way to identify and characterize the site-specific nature of these drugs, as well as a means for testing the potency and tolerable levels of these drugs. This methodology will be invaluable for developing more precise molecular biology tools to utilize for the study and treatment of diseases that are associated with aberrant histone acetylation.

## Figures and Tables

**Table 1:** Summary of effects of tested compounds in vivo and in vitro.

In Vitro	Fold Change in k_cat_						Predominant Zebrafish Phenotype						
	K9	K14	K18	K23	[Drug]								
C646 (activate) 4.02		6.25	1.65	2.75	2.5 µM		C646	6 h	24 h				
C646	0.38	0.07	0.19	0.17	15 µM		1 uM	Normal	Normal				
CTB	0.23	0.74	1.34	1.48	100 µM		2.5 uM	Normal	Normal				
CTPB	0.99	0.99	0.84	1.64	100 µM		5 uM	Normal	Moderate		Garcinol	6 h	24 h
Garcinol	0.38	0.41	0.52	0.40	100 µM		10 uM	Normal	Dead		1 uM	Normal	Moderate
EGCG	0.31	0.45	0.50	0.45	100 µM		50 uM	Normal	Dead		10 uM	Normal	Dead
											25 uM	Dead	Dead
	kcat (×10^−3^ sec) w/drug						CTB	6 h	24 h		75 uM	Dead	Dead
	**K9**	**K14**	**K18**	**K23**			1 uM	Normal	Moderate		150 uM	Dead	Dead
Ctr	7.48	30.62	380.5	145			10 uM	Dead	Dead				
C646	30.17	191.3	629.2	399	2.5 µM		25 uM	Dead	Dead				
CTB	1.71	22.65	508.04	215.17	100 µM		75 uM	Dead	Dead		EGCG	6 h	24 h
CTPB	7.43	30.38	320.69	237.08	100 µM		150 uM	Dead	Dead		1 uM	Normal	Normal
Garcinol	2.82	12.44	197.85	58.32	100 µM						10 uM	Normal	Normal
EGCG	2.34	13.81	189.58	65.07	100 µM						25 uM	Normal	Normal
							CTPB	6 h	24 h		75 uM	Normal	Normal
**In cells**	Fold change in fraction acetylated					**Overall**	1 uM	Normal	Normal		150 uM	Normal	Normal
	**K9**	**K14**	**K18**	**K23**		**Change**	10 uM	Normal	Normal				
C646 (activate) 1.61		1.32	2.31	1.44	2.5 µM	2.7	25 uM	Normal	Normal				
C646 (inhibit) 0.20		0.68	1.10	0.87	50 µM	8.7	75 uM	Normal	Normal				
CTB	0.16	1.02	0.70	0.94	150 µM	9.8	150 uM	Normal	Normal				
CTPB	1.27	0.69	0.59	0.97	150 µM	1.5							
Garcinol	0.001	0.24	0.16	0.40	150 µM	1129.3							
EGCG	0.59	0.92	1.11	1.00	150 µM	0.9							
**In vitro comparison between p300 and CBP (Fold change in k_cat_)**													
	**p300**	**CBP**	**p300**	**CBP**	**p300**	**CBP**	**p300**	**CBP**					
	**K9**		**K14**		**K18**		**K23**		**Drug**				
C646	4.02	0.05	5.38	0.11	6.26	0.54	7.23	0.10	2.5 µM	(0.75 µM for CBP)			
CTB	0.23	0.10	0.74	0.15	1.34	0.02	1.48	0.06	100 µM				
CTPB	0.99	1.03	0.99	0.76	0.84	1.03	1.64	1.11	100 µM				
Garcinol	0.38	0.16	0.41	0.24	0.52	0.13	0.40	0.26	100 µM				
EGCG	0.31	0.54	0.45	0.47	0.50	0.78	0.45	1.18	100 µM				

## Supplementary Materials

The following are available online at https://www.mdpi.com/2073-4409/8/9/1022/s1, Figure S1: Analysis of ligand binding distribution and affinity, and pdb files and a PyMOL session of the binding found in Figure 4.
