# Supplementary material for: Discordant Effects of Putative Lysine Acetyltransferase Inhibitors in Biochemical and Living Systems
Source: Cells. 2019 Sep 2;8(9):1022. doi: 10.3390/cells8091022 (PMC6770547; doi:10.3390/cells8091022)
Supplement: Supplementary file 1 [file cells-08-01022-s001.zip › Supplemental Figure 1, Related to Figure 4.pdf]

Supplemental Figure 1, Related to Figure 4

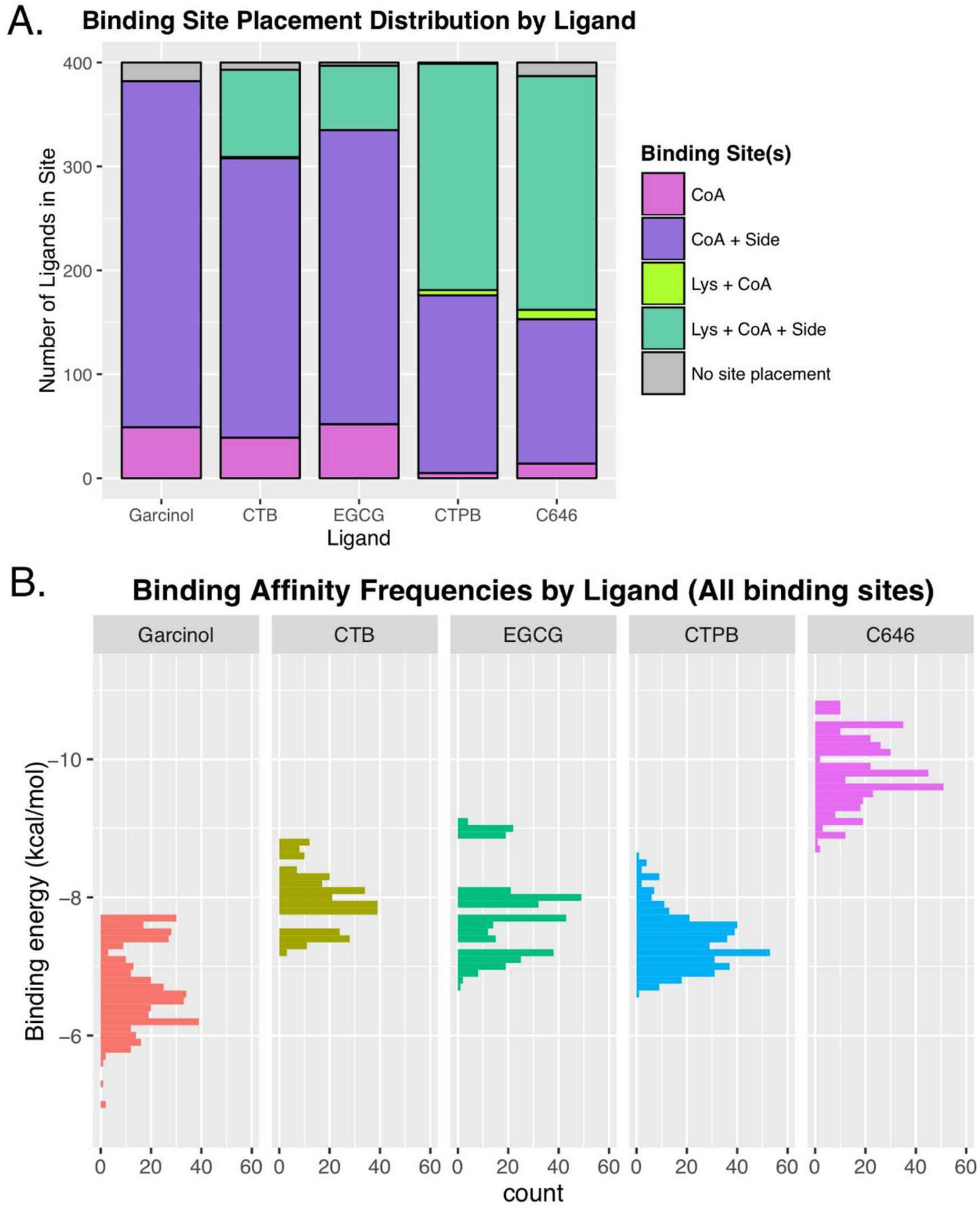

C.

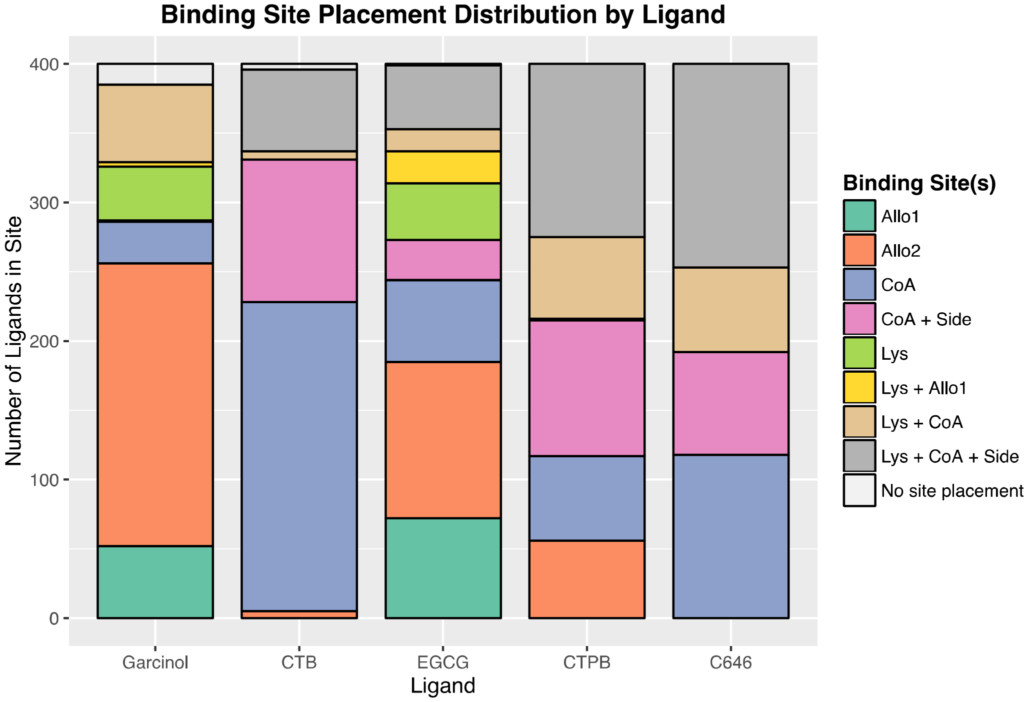

Analysis of ligand binding distribution and affinity. A) Quantitative assessment of the binding site distribution of the 400 ligand poses upon docking to the active site of the KAT domain of p300. Ligands that simultaneously occupied multiple sites were also identified. Interestingly, none of the ligands occupied only the lysine binding pocket. B) Histogram showing the frequency of ligand binding affinities calculated for each docking pose when docket to the active site. C646 had the tightest average binding affinity while garcinol was the least tightly bound, the remaining three ligands having intermediate binding affinities. C) Quantitative assessment of binding site distribution of the 400 ligand poses upon docking to the entire protein.
